# Supplementary material for: Metabolic Profiling of Rheumatoid Arthritis Neutrophils Reveals Altered Energy Metabolism That Is Not Affected by JAK Inhibition
Source: Metabolites. 2022 Jul 15;12(7):650. doi: 10.3390/metabo12070650 (PMC9321732; doi:10.3390/metabo12070650)
Supplement: Supplementary file 1 [file metabolites-12-00650-s001.zip › metabolites-1772442-supplementary.pdf]

Supplementary Methods

Chemotaxis Assay

The chemotaxis assay was carried out in 24-well tissue culture plates (coated with 12mg/mL polyhema to prevent cell adhesion) using hanging chamber inserts with a 3μM porous membrane, as previously described [18]. fMLP (10^{-8}M) was added to RPMI media in the bottom chamber. Neutrophils (10^6/mL) were incubated with or without tofacitinib, baricitinib, or pan-JAK inhibitor I (all 200 ng/mL) for 30 min prior to addition to the top chamber and incubated for 90 min at 37 °C and 5% CO_2. The number of migrated cells after 90 min incubation was measured using a Coulter Counter Multisizer-3 (Beckman Coulter, High Wycombe, UK).

Phagocytosis Assay

Neutrophils (10^6/mL) were incubated with or without tofacitinib, baricitinib or pan-JAK inhibitor I (all 200 ng/mL) for 30 min prior to the addition of FITC-labelled latex beads (1.1 μm diameter) for 30 min (100:1 ratio beads:cells). Cells were centrifuged at 1000g for 3 mins and resuspended in HBSS prior to analysis on a Beckman Coulter CytoFLEX flow cytometer where 10,000 events were analysed.

Supplementary Data

Supplementary Table S1. List of annotated metabolites from neutrophil spectra. This table reports the total number of annotated peaks for each metabolite and the level of identification based on the metabolite reporting standards set out by the metabolomics society. A representative bin was chosen out of all related annotated peaks and brought forward in the subsequent statistical analysis. HMDB = Human Metabolome Database

Metabolite	HMDB ID	Number of Peaks Annotated in Spectrum	MSI Level of Identification	Representative Bin
2 Hydroxyvaleric acid	HMDB0001863	12	2	1.3539 - 1.433
2-Hydoxybutyric acid	HMDB0000008	3	2	0.9106 - 0.9058
2-Hydroxy-3-methylpentaonic acid	HMDB0000317	24	2	0.8962 - 0.8504
3.Hydoxybutyric acid	HMDB0000011	2	2	1.2241 - 1.2168
3-Hydroxyisovaleric.acid	HMDB0000754	2	2	1.2572 - 1.2498
Acetamide	HMDB0031645	1	2	2.0094 - 2.0039
Acetoatic acid	HMDB0000060	1	2	2.2674 - 2.2618
Acetone	HMDB0001659	1	2	2.2385 - 2.2342
Adipic Acid	HMDB0000448	7	2	1.5445 - 1.5401
ADP	HMDB0001341	6	1	8.5451 - 8.5407
ATP	HMDB0000538	9	1	8.2742 - 8.2719

Choline	HMDB0000097	1	2	3.2129 - 3.2046
Cyclic AMP	HMDB0000058	8	1	6.1548 - 6.1498
D-Glucose	HMDB0000122	62	1	3.8952 - 3.8917
D-Glutamic Acid	HMDB0003339	36	2	2.343 - 2.3384
Dimethylamine	HMDB0000087	1	1	2.7313 - 2.735
D-Mannose	HMDB0000169	10	2	3.3712 - 3.3685
Ethanol	HMDB0000108	3	2	1.1995 - 1.195
Formic acid	HMDB0000142	1	2	8.4626 - 8.4562
Glutathione	HMDB0000125	13	2	2.5718 - 2.5614
Glycerol	HMDB0000131	7	2	3.6449 - 3.6424
Glycol	HMDB0001881	2	2	1.1443 - 1.1381
GTP	HMDB0001273	3	2	5.9546 - 5.9465
Indoleactic acid	HMDB0000671	5	2	7.7515 - 7.7414
Inosinic acid	HMDB0000175	5	2	6.1629 - 6.1548
Isobutyric acid	HMDB0001873	1	2	1.1137 - 1.1082
Isopropyl alcohol	HMDB0000863	3	2	1.1847 - 1.1759
Lactic acid	HMDB0000190	6	1	4.1209 - 4.1167
L-Alanine	HMDB0000161	5	1	1.4826 - 1.4768
L-Arginine	HMDB0000517	28	2	1.6403 - 1.6349

L-Aspartic.acid	HMDB0000168	17	1	2.8595 - 2.8542
L-Glutamine	HMDB0000641	26	1	2.4785 - 2.4733
L-Histidine	HMDB0000177	2	1	7.0845 - 7.0758
L-Isoleucine	HMDB0000172	21	1	1.0154 - 1.0059
L-Leucine	HMDB0000687	28	1	0.9695 - 0.9613
L-Lysine	HMDB0000182	28	2	3.0323 - 3.0275
L-Methionine	HMDB0000696	12	1	2.6355 - 2.6264
L-Phenylalanine	HMDB0000159	11	1	7.3427 - 7.3364
L-Proline	HMDB0000162	12	2	3.3052 - 3.3001
L-Serine	HMDB0000187	23	2	3.9558 - 3.9528
L-Threonine	HMDB0000167	8	1	4.2574 - 4.2538
L-Tyrosine	HMDB0000158	9	2	6.9145 - 6.9107
L-Valine	HMDB0000883	13	1	0.9934 - 0.9867
Methyl Acetate	HMDB0015502	1	2	1.9225 - 1.9169
Myo-Inositol	HMDB0000211	14	1	3.2853 - 3.2821
NAD+	HMDB0000902	10	1	9.1575 - 9.1485
NADH	HMDB0001487	18	2	8.2099 - 8.2052
NADP+	HMDB0000217	10	2	8.1921 - 8.184
Phosphocholine	HMDB0001565	1	2	3.2284 - 3.2211

Propionic acid	HMDB0000237	9	2	1.0745 - 1.0659
Pyroglutamic acid	HMDB0000267	30	2	2.5178 - 2.5141
Saccharopine	HMDB0000279	46	2	3.0819 - 3.0742
Sarcosine	HMDB0000271	2	2	2.234 - 2.239
Taurine	HMDB0000251	9	1	3.4164 - 3.4124

Supplementary Table S2. PLS-DA validation table. Percentage score of all assessed metrics in the machine learning model with their standard deviation. UNTR = untreated, JAKi = pan JAK inhibitor I, BARI = Baricitinib, TOFA = Tofacitinib.

	**Accuracy**	**Balanced Accuracy**	**F1 Score**	**Precision**	**Recall**
UNTR 0h	90% (13.7)	90% (13.7)	86.7% (18.3)	100% (0)	80% (27.4)
UNTR 2h	95% (11.2)	95% (11.2)	93.3% (14.9)	100% (0)	90% (22.4)
JAKi	85% (13.7)	85% (13.7)	80% (18.3)	100% (0)	70% (27.4)
BARI	95% (11.2)	95% (11.2)	93.3% (14.9)	100% (0)	90% (22.4)
TOFA	95% (11.2)	95% (11.2)	93.3% (14.9)	100% (0)	90% (22.4)

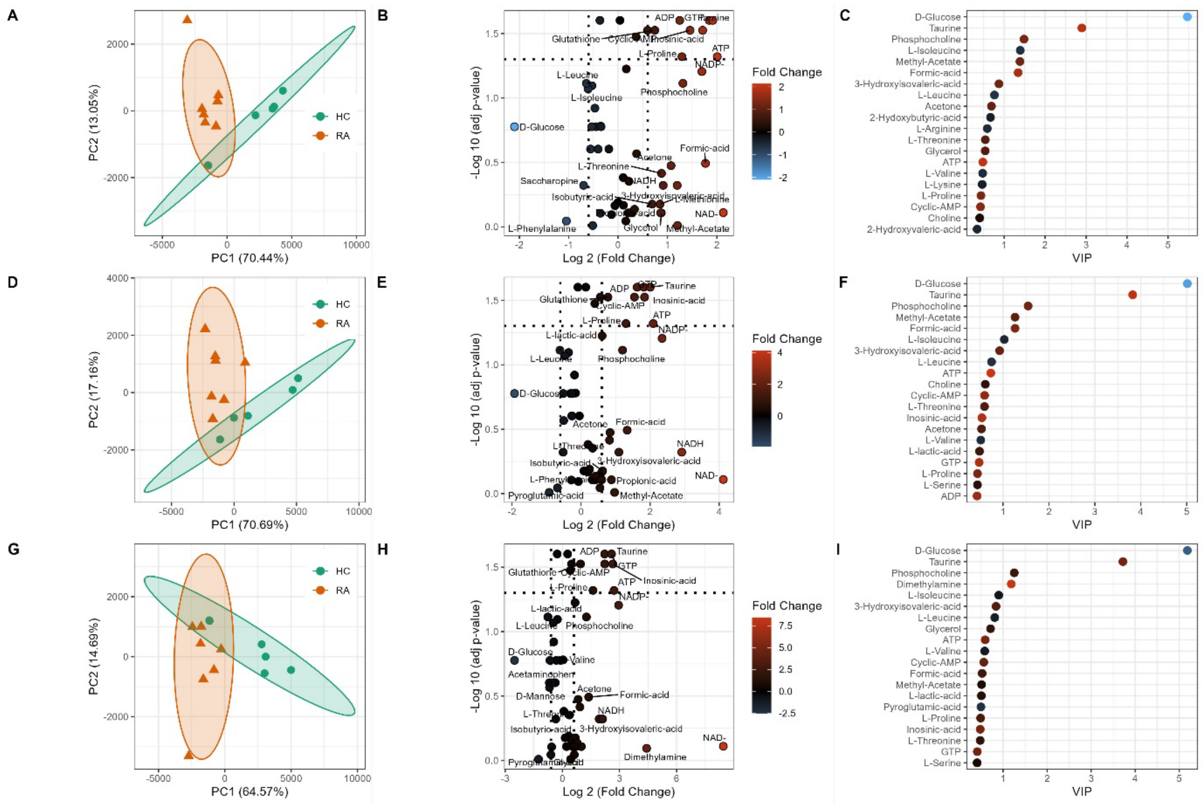

Supplementary Figure S1. Metabolomics differences between RA and HC neutrophils with JAK inhibitors. PCA scores plot of HC and RA neutrophils with pan-JAKi (**A**) Baricitinib (**D**) and Tofacitinib (**G**) showing separation on the first principal component (**PC**). Volcano plot (**B,E,H**) showing metabolites significantly different between HC and RA neutrophils (adj p-value < 0.05) and the log2 fold change (**FC**) for each metabolite as indicated by gradient colour scale provided. Variable importance in projection (VIP) (**C,F,I**) obtained from PLS-DA showing top 20 metabolites for each model.

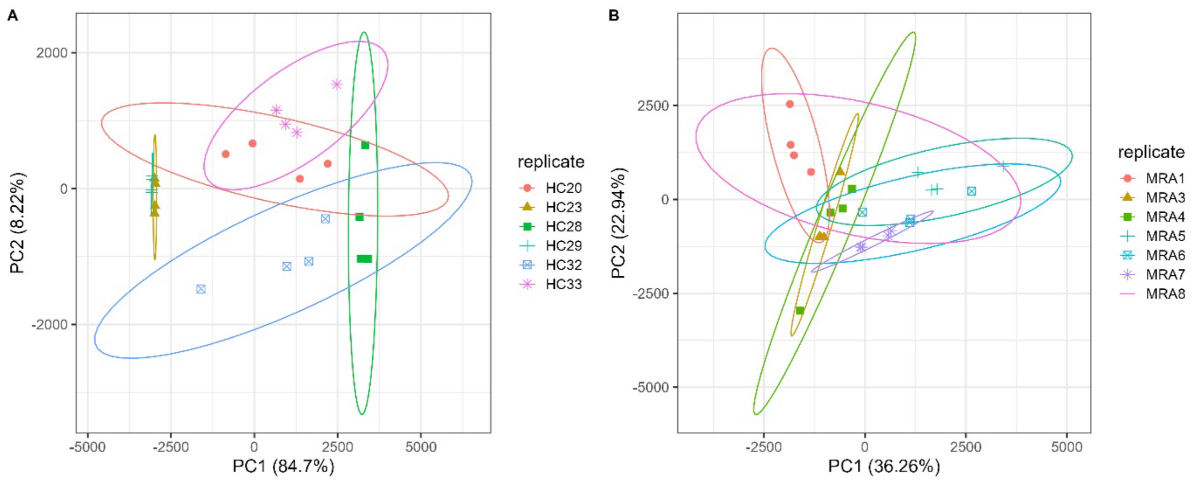

Supplementary Figure S2. PCA showing high between-subject variability of neutrophils treated with JAK inhibitors. (HC = healthy control, RA = rheumatoid arthritis).

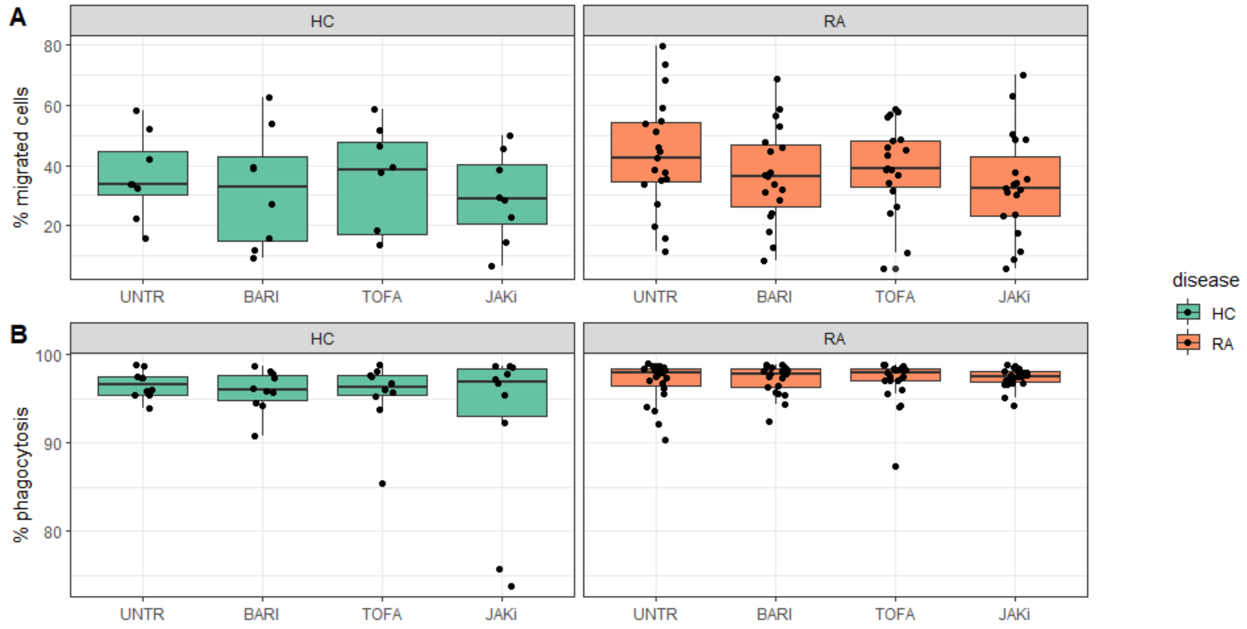

Supplementary Figure S3. Effect of JAK inhibitors on neutrophil migration and phagocytosis. (**A**) Effect of JAK inhibitors on neutrophil chemotaxis towards fMLP. Neutrophils were pre-treated with JAKi for 30 min and then allowed to migrate towards fMLP (10^{-8}M) for 90 min; (**B**) effect of JAK inhibitors on neutrophil phagocytosis. Neutrophils were pre-treated with JAKi for 30 min and then incubated with FITC-labelled latex beads for 30 min. None of the inhibitors tested prevented phagocytosis or cell migration. UNTR = untreated, JAKi = pan JAK

inhibitor I, BARI = Baricitinib, TOFA = Tofacitinib.
